# The Impact of Body Mass Index and Weight Changes on Disability Transitions and Mortality in Brazilian Older Adults

**DOI:** 10.1155/2013/905094

**Published:** 2013-04-04

**Authors:** Flávia Cristina Drumond Andrade, Ahmad Iqmer Nashriq Mohd Nazan, Maria Lúcia Lebrão, Yeda Aparecida de Oliveira Duarte

**Affiliations:** ^1^Department of Kinesiology and Community Health, University of Illinois at Urbana-Champaign, 1206 South 4th Street, Champaign, IL 61820, USA; ^2^Zilber School of Public Health, University of Wisconsin-Milwaukee, 1240 N. 10th Street, Milwaukee, WI 53205, USA; ^3^School of Public Health, Departament of Epidemiology, University of São Paulo, Avenida Dr. Arnaldo, 715 Pinheiros, 01246-904 São Paulo, SP, Brazil; ^4^School of Nursing, Department of Medical-Surgical Nursing, University of São Paulo, Avenida Dr. Enéas de Carvalho Aguiar, 419 3o. andar, Sala 318, Cerqueira Cesar, 05403-000 São Paulo, SP, Brazil

## Abstract

The aim of this study was to examine the association between body mass index and weight changes on disability transitions and mortality among Brazilian older adults. Longitudinal data from the Health, Well-Being, and Aging in Latin America and the Caribbean Study conducted in São Paulo, Brazil (2000 and 2006), were used to examine the impact of obesity on disability and mortality and of weight changes on health transitions related to disability. Logistic and multinomial regression models were used in the analyses. Individuals who were obese were more likely than those of normal weight to have limitations on activities of daily living (ADL), instrumental activity of daily living (IADL), and Nagi's limitations. Obesity was associated with higher incidence of ADL and IADL limitations and with lower recovery from Nagi's limitations. Compared to those who maintained their weight, those who gained weight experienced higher incidence of ADL and Nagi's limitations, even after controlling for initial body mass index. Higher mortality among overweight individuals was only found when the reference category was “remaining free of Nagi limitations.” The findings of the study underline the importance of maintaining normal weight for preventing disability at older ages.

## 1. Introduction

Brazil is among the 25 countries in the world with the fastest aging rates [1, 2]. In 1950, 2.6 million (4.9%) Brazilians were older than 60 years of age, and this number has increased to 20.6 million people (10.8%) according to the 2010 census [3]. Improvements in medical care and living standards have been shown to translate into higher life expectancy. In 1950, for example, life expectancy at birth in Brazil was 50.9 years, but the figure increased to 72.2 years in 2010 [3]. However, the number of disabled people is expected to increase in the coming years, given the rapid growth rate of the elderly population and the rise in the prevalence of obesity and chronic diseases [4].

Fast changes in the population's nutritional intake that have occurred in Brazil in recent decades [5] have resulted in an increase in the prevalence of obesity [6, 7]. In the past three decades, obesity rates in Brazil tripled among men and almost doubled among women [6]. With the exception of higher-income urban women [8], the prevalence of obesity is higher among women than among men.

Few studies focus on the impact of BMI on mortality and disability in the Latin American and the Caribbean (LAC) region. Based on the baseline for the Health, Well-Being, and Aging in Latin America and the Caribbean Study (SABE), Al Snih and colleagues [4] showed that obese individuals were 1.6 times more likely to face difficulties performing activities of daily living (ADL) than those with normal BMI [4]. Corona and colleagues [9] found that older adults who were underweight (BMI ≤ 23 kg/m^2^) and obese (BMI ≥ 30 kg/m^2^) were more likely to develop limitations on instrumental activity of daily living (IADL), but their analysis did not explore BMI associations with mortality or recovery from disability. Monteverde and colleagues [10] found that, based on relative cutoffs (quintiles), heavier older adults in Mexico face higher mortality risks than those in the United States. However, when BMI was categorized following the traditional World Health Organization cutoffs, no excess mortality was found among overweight and obese subjects [10]. In fact, coefficients for overweight and obesity were not in the expected direction [10]. However, none of these studies jointly examined the association between BMI, disability transitions, and mortality. This paper addresses this gap in the literature. We examine the association between body mass index and weight changes on disability transitions and mortality. 

### 1.1. Association between BMI and Disability

Obesity has been associated with higher prevalence of disability in cross-sectional and longitudinal studies [4, 11–13]. This positive association has been found among middle- and older-aged adults [13–15], and it appears that this association has not changed over time [16, 17]. Additionally, the association between body mass index (BMI) and disability is strongest among those who are underweight (BMI < 18.5 kg/m^2^) and among obese subjects (BMI > 30 kg/m^2^) [4, 11]. Obese women face higher prevalence of mobility impairment than men [13]. In the United States, severe functional limitations are higher among older adults who gain or lose weight after age 50 compared to those with stable weight [18]. Emerging evidence supports the proposition that BMI is an important predictor of the onset of mobility limitations [13]. Older adults in the United States who gain weight over time have higher incidence of mobility limitations than those who maintain their weight [19].

### 1.2. Association between BMI and Mortality

Large systematic reviews have shown that the relationship between BMI and mortality seems to follow a J-shaped (sometimes U-shaped) curve [17, 20–22]. All-cause mortality appears to be lowest at BMI levels between 20 and 24.9 kg/m² [20, 22], with obese individuals facing higher mortality risks than their normal-weight counterparts [20, 22, 23]. Recent studies, however, show that overweight individuals have higher life expectancy than individuals of normal weight or those who are obese [12], whereas other studies have an L-shape (see [24], for a short review). Higher mortality at low levels of BMI has been associated primarily with lung cancer and respiratory diseases [22]. At older ages, results from a systematic review and meta-analysis indicated that BMI in the overweight range of 25–29.9 kg/m² is not associated with increased mortality [25], whereas other studies have shown that the burden of obesity on mortality seems to be reduced or eliminated among older adults [11, 12, 26–30]. 

The use of BMI categories has been criticized for not reproducing well the complexities of the BMI and mortality relationship [31–33]. These authors have suggested that the use of alternative models to assess this association. Gronniger [33] used semiparametric models and found that men in the mild-obese category (BMIs of 30–35 kg/m^2^) had similar mortality than those of normal weight, but among women BMI levels above 27 kg/m^2^ were associated with higher mortality in the U.S. Wong and colleagues [31] used multivariable fractional polynomials to explore the association between BMI and mortality in a sample of adults in the UA They found that the best fitting model contained the powers −1 and −2 for BMI [31]. Their results indicated that the nadir of the BMI mortality curve was in the normal range for women but overweight range for men [31]. Zajacova and Burgard [32] used generalized additive models and found that the nadir was at BMIs 23 to 26, which is also in the normal overweight range. However, they point out that there are important differences depending on the cause of death. For example, the association between BMI and diabetes mortality increases monotonically, but, for all-cause mortality, it followed more a V-shape [32]. However, even though these alternative models often provide better fit to the data, the results of these studies are often interpreted making references for traditional cutoffs as they are more easily understood by the general audience and health practitioners. The use of BMI and BMI categories have also been criticized because it can be related to underlying health status [17]. For example, individuals may be underweight based on their BMI because they have health conditions such as cancer, thyroid problems, infectious, or digestive diseases that lead to low body weight. One approach to address this limitation has been to take into account body weight changes [17].

Our study uses data a large cohort study conducted in São Paulo, Brazil, to examine the association between BMI and body weight changes on disability transitions and mortality, while controlling for a series of demographic, socioeconomic, and health determinants. We investigate these associations on three types of disability (activities of daily living, instrumental activities of daily living, and Nagi's limitations) transitions.

## 2. Materials and Methods

Data from the two waves (2000 and 2006) of the SABE cohort study conducted in São Paulo, Brazil, were used in this study. SABE is a multicenter survey with respondents in seven major cities throughout LAC countries that have been investigating the health and well-being of older adults (age 60 and over). The study was approved by the Institutional Review Boards at the collaborating institutions [34, 35], and the participants provided consent to have their data used for research purposes.

The baseline sample was obtained using a two-stage stratified sampling based on the 1995 National Household Survey master sampling frame. The data in the first wave were collected in two stages. The first stage was a household interview conducted by a single interviewer using a standardized questionnaire that included several questions about the living conditions and health status of the subjects. The second stage of data collection consisted of a household visit by a pair of interviewers who completed anthropometric and physical-performance measurements. At baseline, the response rates reached 84.6% in São Paulo. In the first stage, information on 2,143 individuals was collected. Additional characteristics of the baseline data collection process have been described elsewhere [36–38].

In 2006, to reestablish contact, trained interviewers visited the addresses and neighborhoods of surviving participants from the 2000 survey. For those not found during these visits, interviewers used the additional contact information collected at baseline (e.g., telephone numbers of children or other relatives) to obtain further information about the subjects' current location. In 2006, researchers collected data via face-to-face interviews using a standardized questionnaire. The 2006 questionnaire was very similar to the one used in 2000 but included additional questions that complemented the previous study. Vital statistics records were used to identify subjects who had died between 2000 and 2006. The search was based on the names, sex, dates of birth, and addresses listed in the 2000 database.

Of the 2,143 participants in the first wave of SABE São Paulo, 355 (16.6%) had missing data on selected variables. Most of them (*n* = 347) had missing data on BMI measure. Those with missing data were older (75.1 years) than those with complete data (72.9 years) (*P* = 0.0001), but there were no sex differences. The prevalence of all measures of disability was higher among those with missing data (*P* < 0.001). The final sample is composed of 1,788 individuals, with a subset of 961 with weight change included in the analyses. There were 473 participants who died between the baseline and the followup in 2006.

### 2.1. Measures

Self-reported disability in six ADL measures (dressing, bathing, eating, getting in and out of a bed, toileting, and getting across a room) were used to measure disability. Individuals were given the following introduction: “Here are a few everyday activities. Please tell me if you have any difficulty with these because of a health problem. Exclude any difficulties you expect to last less than three months.” After this introduction, they were asked “Do you have difficulty…?” And the possible answers were: “yes,” “no,” “does not know,” and “no response” for each one of the six ADL measures. Participants who answered “does not know” and “no response” were classified as missing. IADL questions followed the ADL ones. No additional introduction was made. Individuals were asked “Do you have difficulty…?” The IADL items included were preparing a hot meal, managing money, shopping, using of transportation within the community, ability to use the telephone, and responsibility for one's own medications. The possible answers were “yes,” “no,” “cannot do it,” “does not know,” and “no response.” Those who answered “cannot do it” were classified as having difficulty performing the activity, whereas those answering “does not know” and “no response” were classified as missing. The Nagi physical performance measure included lifting or carrying objects that weighed five kilograms or more; lifting a coin; pulling or pushing a large object, such as a living-room chair; stooping, kneeling, or crouching; and reaching or extending the arms above shoulder level. Each of the three disability measures was converted into binary form, in which respondents scored “0” if they did not indicate any limitations and “1” if they reported having difficulty performing one or more activity in the scale.

Body weight and height were measured without shoes and with light clothing by trained examiners. BMI was calculated as kg/m^2^. Four BMI categories were defined according to the criteria adopted by the Pan American Health Organization for the SABE study [38]: underweight (BMI ≤ 23.0), normal (BMI > 23 and < 28), overweight (BMI ≥ 28.0 and < 30), and obese (BMI ≥ 30). Change in BMI was calculated as BMI in 2006 minus the BMI at baseline. This difference was divided by the baseline BMI and then recoded into three categories: (a) an increase of 5% or more; (b) a decrease of 5% or more; and (c) changes within 5% of the baseline weight (reference category) [19, 39].

The following sociodemographic characteristics were included in the analysis: age (in years), gender, smoking status (never, former, or current smoker), schooling (in years of formal education), and household arrangement (living alone or accompanied). All regression analysis also included a measure of number of chronic conditions at the baseline. Health status based on the number of self-reported chronic conditions included diabetes, hypertension, cardiovascular disease, stroke, cancer, arthritis, and osteoporosis. 

STATA S.E. 12.1 for Windows (StataCorp, College Station, TX) was used for all the statistical analyses. Descriptive statistics were conducted first. Weighted logistic regressions were then used to assess the influence of BMI on disability prevalence. Multinomial logistic regressions were used to assess the influence of BMI on disability transitions and mortality. For those free of disability, four outcomes were considered in the multinomial logistic regressions: remained free of disability (reference category), became disabled (incidence), died, or were lost to followup. For those who were disabled at the baseline, four outcomes were included in the multinomial logistic regressions: remained disabled (reference category), recovered from disability, died, or were lost to followup. Multinomial logistic regressions were used to analyze the role of weight change on health transitions as discussed above, excluding mortality, as we do not have information on weight change prior to death in between waves. 

In the baseline, there were 1,420 individuals free of ADL and 368 individuals with ADL. In 2006, among those free of ADL, 606 individuals had remained free of ADL, 226 had developed ADL, 329 had died, and 259 were lost in the followup or had missing data on ADL status. Among those who had ADL in the baseline, 99 remained with ADL, 75 recovered from ADL, 144 died and 50 were lost in the followup or had missing data on ADL in 2006. For IADL limitations, 1,207 were free of IADL, and 581 had IADL in the baseline. Among those who were free of IADL in the baseline, 491 remained free of limitations, 257 developed IADL, 230 had died, and 229 were lost in the followup or had missing data on IADL in the second wave. Among those with IADL in the baseline, 220 remained with IADL, 36 recovered from IADL, 243 had died, and 82 were lost in the followup or had missing data on IADL status in 2006. Regarding the Nagi, 654 participants were free of it in 2000, and 1,134 had at least one Nagi limitation. Among those free of Nagi, 192 remained free of it, 210 developed Nagi, 129 died, and 123 were lost in the followup or had missing data on the Nagi variable in 2006. Among those who had at least one Nagi limitation in 2000, 539 remained with Nagi's limitations, 70 recovered from Nagi's limitations, 344 died, and 181 were lost in the followup or had missing data on Nagi's limitations in the second wave.

## 3. Results

In the final sample, 23.4% were underweight, 43.3% had normal weight, 12.4% were overweight, and 21.1% of the participants were classified as obese.  Table 1 presents the prevalence estimates of disability according to measures of ADL, IADL, and Nagi's limitations by sex and BMI category at baseline. Prevalence of ADL and Nagi's limitations was highest among obese individuals, whereas prevalence of IADL was highest among underweight older adults. Weighted estimates indicated that 16.7% of the sample in São Paulo had difficulty performing at least one ADL. Prevalence of IADL reached 24.4%, and most (57.8%) of the older Brazilian adults reported Nagi's limitations. In logistic regressions, after adjusting for age and sex (not shown), individuals who were underweight did not differ from those of normal weight on their likelihood of reporting having ADL, IADL, or Nagi in the baseline. Obese individuals were more likely than normal weight participants to report having at least one ADL (OR = 1.8, 95% CI = 1.2, 2.6) and Nagi's limitations (OR = 2.5, 95% CI = 1.8, 3.6). There were no statistical differences between normal weight and obese participants regarding IADL prevalence at baseline. There were no statistical differences between normal and overweight subjects regarding ADL and IADL prevalence at baseline, but overweight individuals were more likely than those of normal weight to have Nagi's limitations (OR = 1.6, 95% CI = 1.1, 2.2). Women were more likely than men to report having ADL, IADL, and Nagi's limitations at baseline (*P* < 0.05).

Table 2 shows the multinomial logistic regression results of the disability transitions and mortality between 2000 and 2006 among those who were free of disability in the baseline. Compared to normal weight individuals, obese individuals were more likely to develop ADL (RRR = 2.1) and IADL (RRR = 2.4), whereas individuals who were underweight were more likely to develop IADL (RRR = 1.9). Mortality risks were higher among those who were overweight (RRR = 2.5) compared to those of normal weight in the Nagi model in which the reference category was remaining free of Nagi's limitations. For all measures of disability, the risk of becoming disabled increased with age. As expected, older age was associated with higher mortality. Women were more likely to develop ADL and Nagi's limitations, but not IADL, between waves. Women had lower mortality in the ADL and IADL models. Higher number of chronic conditions was associated with higher mortality and higher incidence of ADL and IADL. 

Table 3 shows the multinomial logistic regression results of the disability transitions and mortality between 2000 and 2006 among those who had disability in the baseline. Being obese was also associated with lower recovery from Nagi (RRR = 0.46) versus remaining with at least one Nagi limitation. Older age and higher number of chronic conditions were negatively associated with recovery.

In the last set of analyses, we focus on the role of weight gain between waves on disability transitions (Table 4). The analyses are restricted to those who have survived between waves. The results presented in Table 4 indicate that those who gained weight between waves were more likely to develop ADL (RRR = 2.3) and Nagi's limitations (RRR = 2.2) than those who maintained their weight, even after controlling for initial BMI categories and other covariates. Higher age was also associated with higher incidence of disability. Women faced higher incidence of ADL (RRR = 1.8) and Nagi (RRR = 2.4) than men. Obesity was associated with higher incidence of ADL and IADL. Underweight individuals were more likely to develop IADL. Individuals with more chronic conditions also faced higher incidence of ADL and IADL limitations. When the analyses focused on those who had disability in the baseline, we found that weight gain was associated with lower recovery from ADL (RRR = 0.18). Older age was negatively associated with recovery from disability. A higher number of chronic conditions were associated with lower recovery of ADL and Nagi. Obesity was negatively associated with recovery from Nagi's limitations. 

## 4. Discussion

Most previous studies have focused on the association between BMI and disability [4, 9, 13, 15, 16] or BMI and mortality [10, 20–29, 31–33], but few have analyzed the effect of BMI on both disability and mortality [11, 12, 14, 30]. Using three disability measures and data from a large cohort study, this study contributes to the literature by exploring the impact of BMI and weight changes on disability status transitions and on mortality. This study confirmed the negative effects of obesity on disability in São Paulo, Brazil. Higher levels of Nagi's limitations were also found among those who were overweight at baseline. Most longitudinal studies have found that obese older adults are more likely to have experienced incidence of disability in the followup than those of normal weight [13, 14, 40], and our study confirmed these findings. Older adult Brazilians who were obese at baseline faced higher risks of becoming disabled with ADL or IADL limitations compared to those of normal weight. However, being overweight was not associated with higher incidence of disability after controls were included in the analysis, which is consistent with previous findings [41]. In terms of recovery, we also found that obese individuals were less likely to recover (versus remaining disabled in the followup), as other studies have also found [40].

There is growing interest in the role of weight changes on health transitions [13, 18, 19, 42]. Studies have shown that weight gain in older adults is associated with decreased physical function and role limitations [18, 19]. We found similar findings in which older adults who gained weight between waves were more likely to develop ADL and Nagi's limitations than those who maintained their weight, even after controlling for initial BMI categories. Al Snih and colleagues [19] also reported higher ADL incidence among individuals who had weight gain of more than 5% between waves. Studies have reported contradictory findings related to weight loss. Some studies have indicated that weight loss is associated with improvements in mobility and functioning [13], whereas others have reported increased ADL disability [19]. Weight-loss therapy among obese older adults seems to be beneficial for improving quality of life and physical functioning [43]. Ritchie and colleagues [42] found that intentional weight loss was not associated with functional decline; however, those who unintentionally lost weight faced higher rates of functional decline, regardless of the initial BMI. In our study, we found no differences between those who lost weight and those who maintained weight on disability transitions after controlling for initial BMI. Given the lack of data on intent related to weight changes, further studies are necessary to explore the impact of weight change on mortality and disability in Latin American countries.

In additional analyses (not shown and available upon request), we have explored additional models to test whether BMI and weight changes influence changes in the number of disabilities over time. We found that obesity was associated with the increases in the number of Nagi's limitations. Weight loss and weight gain were associated with an increase in the number of ADL and Nagi's limitations over time. Changes in the number of IADL limitations were not statistically associated with BMI categories or weight changes. As expected, older age was associated with the increases in the number of ADL, IADL, and Nagi's limitations over time. A higher number of chronic conditions were also associated with an increase in the number of ADL, IADL and Nagi's limitations over time. Being female was also positively associated with increases in the number of Nagi limitations. We also tested fractional polynomial models following the approach suggested by Wong and colleagues [31] to examine the relationship between BMI, disability and mortality (results available upon request), and our main conclusions remain the same, which indicates that findings are robust to different model specifications. 

The only mortality differential by BMI categories was found among overweight participants who were more likely to die than to remain free of Nagi's limitations. In further analyses (not shown), results from a logistic regression that controlled for the same covariates included in this study, revealed no differences in mortality among underweight, normal weight, overweight, and obese participants. This is consistent with previous studies suggesting that the association between BMI and mortality becomes less U-shaped at older ages [44], and others that suggest that higher BMI may not be detrimental for mortality at older ages [12]. Monteverde and colleagues [10] also did not find statistical differences in mortality among older adults between higher BMI categories (overweight and obese) and normal subjects when using traditional BMI cutoffs, though they reported statistical differences when BMI was categorized in relative terms. However, some studies allude to the fact that the association between BMI and mortality is differential between individuals who are healthy versus those with chronic conditions [45]. The obesity paradox literature indicates that excess weight is actually protective among patients with chronic disease [45]. In our sample, there were no mortality differentials by BMI categories among those with chronic conditions, but overweight individuals free of chronic conditions had lower mortality than those of normal weight (results available upon request). 

Our findings also contribute to a growing debate in the field about whether greater life expectancy implies better health for the expanding surviving elderly female population in Latin America [37, 46–51]. We found that Brazilian women experience higher levels of disability than men, which is consistent with previous studies [52, 53]. Previous studies have indicated that Brazilian women face lower mortality than their male counterparts [37, 54], and this study confirms these findings. 

Aging is related to the increase of fat mass, and there is growing evidence of the detrimental impact of obesity on disability at older ages. There is evidence as well that changes in lifestyle, such as walking, have positive effects on preventing mobility limitations [55]. A large proportion of older adults, however, do not engage in physical activity. In a study based on an urban sample in Brazil, for example, 71% of older adults reported living sedentary lives [56]. When asked about neighborhood characteristics related to concerns of leaving home to go out, most (78%) reported fear of being robbed, while almost half (48.2%) said that they were afraid of falling because of sidewalk defects [56]. Fear of falling due to poor sidewalk conditions was associated with a 62% increase in the expected number of ADL conditions [56]. Therefore, investments aimed at improving urban infrastructure and safety may be effective in addressing the health conditions of older adults in Brazil.

This study advances the literature on the impact of body weight and body weight changes on disability and mortality. This study, however, has some limitations. First, the data used in the study on disability measures were self-reported. Although this could be a possible source of bias, methodological studies have shown that self-reported data on functional disability are consistent with medical diagnoses [57]. Second, the use of BMI as a measure for body weight composition among older adults is very controversial as it does not take into account body fat distribution [17]. In addition, BMI at baseline can be associated with health status [16, 17]. Therefore, it is important to control for weight changes, which we accomplished in this study. Some authors have argued that waist circumference or waist-to-hip ratio could be better predictors of disability and mortality [10]; however, most studies to date have focused on the use of BMI and the categories used here. Other scholars have indicated that, at least for developed countries, information on BMI, waist circumference, or waist-to-hip ratio do not necessarily improve prediction of mortality due to cardiovascular disease; instead, they suggest using information on systolic blood pressure, diabetes status, and lipids when those are available [58]. And still others argue that—in addition to BMI—waist circumference and waist-to-hip ratio can be useful in better understanding mortality risks [59, 60]. In Brazil, as in other developing countries, data on blood pressure and lipids are often lacking, so the use of anthropometric measures, such as waist circumference, may improve our understanding of the impact of body composition changes on mortality and disability. Third, the first wave of SABE focuses on the civilian population not residing in institutions. As a result, estimates may be biased if one expects institutionalized individuals, particularly those residing in nursing homes, to be more likely to have a higher prevalence of disability than the noninstitutionalized population. However, because the institutionalized population in Brazil is relatively small [61], this possible bias is likely not to be very significant.

## 5. Conclusion

This study confirms previous studies that have found obesity to be associated with increased disability in Brazilian older adults. Historically, Brazil has mainly been concerned with curbing malnutrition; however, in recent years, new policies have targeted the marketing of highly processed and unhealthy foods [5]. Owing to the fact that obesity rates in Brazil have been increasing drastically for the past three decades [6], our findings have important implications for policy makers in Brazil with regard to curbing disability risk by promoting the use of effective preventive measures to reduce body weight, thereby making healthy aging a reality.

## Figures and Tables

**Table 1:** Prevalence of ADL, IADL, and Nagi's limitations, by sex and BMI categories, São Paulo, 2000 (weighted estimates).

	Total	Underweight	Normal	Overweight	Obese	*P*
Total	*n* = 1,788	*n* = 419	*n* = 775	*n* = 217	*n* = 377	
ADL	16.7	14.7	13.9	18.2	23.4	∗∗
IADL	24.4	28.9	21.0	25.0	26.7	∗∗
Nagi	57.8	52.7	50.7	61.5	74.9	∗∗∗
Females	*n* = 1,062	*n* = 212	*n* = 415	*n* = 130	*n* = 305	
ADL	19.8	14.8	17.2	22.2	25.4	∗∗
IADL	30.5	36.5	26.7	33.8	30.4	
Nagi	67.2	59.7	60.5	73.9	78.3	∗∗∗
Males	*n* = 726	*n* = 207	*n* = 360	*n* = 87	*n* = 72	
ADL	12.3	14.5	10.3	13.4	15.7	
IADL	15.6	19.9	14.6	13.9	12.7	
Nagi	44.1	44.4	39.6	45.9	61.8	∗∗

ADL: activities of daily living; IADL: instrumental activities of daily living.

***P < 0.001; **P < 0.05; *P < 0.10.

**Table 2:** Relative risk ratios of the impact of body mass index categories on disability transitions and mortality among those who were free of disability in the baseline, São Paulo, Brazil—2000–2006.

Variables	ADL		*P*	IADL		*P*	NAGI		*P*
	RRR^a^	95% CI		RRR	95% CI		RRR	95% CI	
*Incidence of disability *									
Age	1.10	[1.07,1.13]	∗ ∗ ∗	1.11	[1.08,1.14]	∗ ∗ ∗	1.05	[1.01,1.09]	∗ ∗
Female	1.70	[1.06,2.73]	∗	1.48	[0.86,2.54]		2.37	[1.40,4.02]	∗ ∗
Smoking status									
Former smoker	0.94	[0.60,1.46]		1.15	[0.68,1.94]		1.36	[0.80,2.32]	
Current smoker	1.58	[0.88,2.85]		1.52	[0.73,3.16]		0.93	[0.52,1.65]	
Number of chronic conditions	1.56	[1.33,1.83]	∗ ∗ ∗	1.33	[1.12,1.58]	∗ ∗	1.23	[0.94,1.62]	
Schooling	0.95	[0.86,1.04]		0.91	[0.82,1.00]		0.88	[0.79,0.98]	∗
Live alone	0.81	[0.50,1.31]		0.61	[0.35,1.06]		0.93	[0.39,2.24]	
BMI categories									
Underweight	1.16	[0.66,2.01]		1.92	[1.21,3.03]	∗ ∗	1.03	[0.56,1.89]	
Overweight	0.93	[0.49,1.75]		1.57	[0.84,2.96]		1.86	[0.89,3.87]	
Obese	2.07	[1.21,3.57]	∗ ∗	2.42	[1.65,3.53]	∗ ∗ ∗	1.19	[0.63,2.26]	
*Mortality *									
Age	1.11	[1.08,1.14]	∗ ∗ ∗	1.13	[1.10,1.16]	∗ ∗ ∗	1.11	[1.07,1.16]	∗ ∗ ∗
Female	0.60	[0.38,0.95]	∗	0.41	[0.25,0.65]	∗ ∗ ∗	0.68	[0.34,1.36]	
Smoking status									
Former smoker	1.25	[0.82,1.90]		1.17	[0.67,2.03]		0.90	[0.46,1.77]	
Current smoker	2.89	[1.66,5.03]	∗ ∗ ∗	2.69	[1.36,5.33]	∗ ∗	2.68	[1.13,6.37]	∗
Number of chronic conditions	1.40	[1.16,1.68]	∗ ∗ ∗	1.41	[1.17,1.69]	∗ ∗ ∗	1.82	[1.25,2.64]	∗ ∗
Schooling	0.95	[0.89,1.02]		0.94	[0.87,1.02]		0.93	[0.82,1.07]	
Live alone	1.01	[0.62,1.63]		1.16	[0.64,2.12]		1.36	[0.66,2.79]	
BMI categories									
Underweight	1.21	[0.79,1.85]		1.22	[0.71,2.09]		1.87	[0.95,3.66]	
Overweight	1.30	[0.78,2.14]		1.41	[0.80,2.48]		2.50	[1.01,6.15]	∗
Obese	1.07	[0.65,1.75]		1.29	[0.77,2.18]		0.68	[0.22,2.07]	
*Lost to followup or missing disability status in 2006 *									
Age	1.03	[1.00,1.05]	∗	1.05	[1.02,1.08]	∗ ∗ ∗	1.03	[0.99,1.08]	
Female	1.38	[0.99,1.94]		1.24	[0.84,1.83]		2.19	[1.37,3.52]	∗∗
Smoking status									
Former smoker	1.36	[0.90,2.06]		1.54	[1.00,2.38]		2.01	[1.10,3.70]	∗
Current smoker	1.05	[0.60,1.84]		1.02	[0.53,1.98]		1.32	[0.61,2.89]	
Number of chronic conditions	1.12	[0.96,1.31]		1.12	[0.94,1.34]		1.22	[0.94,1.58]	
Schooling	1.02	[0.92,1.13]		1.01	[0.91,1.12]		0.99	[0.85,1.16]	
Live alone	1.37	[0.84,2.22]		1.42	[0.84,2.40]		0.93	[0.38,2.30]	
BMI categories									
Underweight	1.58	[0.97,2.59]		2.07	[1.24,3.45]	∗ ∗	1.44	[0.70,2.98]	
Overweight	1.25	[0.69,2.26]		1.33	[0.78,2.26]		1.41	[0.59,3.35]	
Obese	1.36	[0.87,2.12]		1.55	[1.02,2.37]	∗	1.32	[0.56,3.14]	
*N*	1,420			1,207			654		
BIC′	−104.55			−82.451			47.73		

ADL: activities of daily living; IADL: instrumental activities of daily living; RRR: relative risk ratio; CI: confidence interval; BMI: body mass index.

^a^Remaining free of disability is the reference category. Normal weight is the reference category for BMI, living accompanied is the baseline category for household arrangement, and never smoked is the reference category for smoking status.

***P < 0.001; **P < 0.05; *P < 0.10.

**Table 3:** Relative risk ratios of the impact of body mass index categories on disability transitions and mortality among those who had disability in the baseline, São Paulo, Brazil—2000–2006.

Variables	ADL		*P*	IADL		*P*	NAGI		*P*
	RRR^a^	95% CI		RRR	95% CI		RRR	95% CI	
*Recovery from disability *									
Age	0.93	[0.88,0.97]	∗∗	0.92	[0.87,0.98]	∗∗	0.96	[0.92,1.00]	∗
Female	0.65	[0.25,1.73]		0.39	[0.13,1.17]		0.56	[0.30,1.07]	
Smoking status									
Former smoker	0.39	[0.11,1.33]		1.04	[0.42,2.57]		1.54	[0.72,3.27]	
Current smoker	1.43	[0.49,4.18]		0.55	[0.11,2.79]		1.01	[0.41,2.49]	
Number of chronic conditions	0.72	[0.52,0.99]	∗	0.93	[0.68,1.27]		0.59	[0.44,0.79]	∗∗∗
Schooling	1.03	[0.83,1.27]		0.99	[0.76,1.28]		1.05	[0.89,1.25]	
Live alone	0.55	[0.17,1.74]		1.11	[0.31,3.92]		3.24	[1.59,6.57]	∗∗
BMI categories									
Underweight	0.37	[0.10,1.34]		0.14	[0.01,1.45]		0.62	[0.25,1.58]	
Overweight	1.01	[0.35,2.97]		0.80	[0.22,2.88]		0.82	[0.29,2.28]	
Obese	0.48	[0.21,1.08]		0.77	[0.28,2.17]		0.46	[0.22,0.97]	∗
*Mortality *									
Age	1.12	[1.07,1.18]	∗∗∗	1.07	[1.04,1.10]	∗∗∗	1.10	[1.08,1.12]	∗∗∗
Female	0.40	[0.18,0.90]	∗	0.65	[0.30,1.38]		0.48	[0.30,0.77]	∗∗
Smoking status									
Former smoker	1.33	[0.57,3.09]		1.75	[0.92,3.34]		1.75	[1.13,2.69]	∗
Current smoker	3.35	[1.06,10.64]	∗	3.09	[1.35,7.07]	∗∗	2.30	[1.29,4.09]	∗∗
Number of chronic conditions	0.87	[0.67,1.14]		0.97	[0.79,1.19]		0.98	[0.86,1.13]	
Schooling	0.98	[0.82,1.17]		1.04	[0.93,1.16]		0.95	[0.89,1.02]	
Live alone	1.03	[0.42,2.50]		0.88	[0.53,1.45]		1.23	[0.77,1.97]	
BMI categories									
Underweight	0.97	[0.44,2.12]		1.28	[0.74,2.21]		0.9	[0.57,1.44]	
Overweight	0.35	[0.11,1.14]		0.75	[0.41,1.39]		0.79	[0.45,1.38]	
Obese	0.54	[0.22,1.35]		0.66	[0.36,1.19]		0.78	[0.51,1.22]	
*Lost to followup or missing disability status in 2006 *									
Age	1.00	[0.94,1.06]		1.00	[0.96,1.04]		1.01	[0.97,1.04]	
Female	0.50	[0.18,1.39]		1.40	[0.48,4.08]		0.93	[0.54,1.60]	
Smoking status									
Former smoker	0.40	[0.13,1.21]		0.69	[0.23,2.04]		1.09	[0.60,1.99]	
Current smoker	0.62	[0.08,4.53]		0.86	[0.30,2.48]		0.66	[0.36,1.19]	
Number of chronic conditions	0.73	[0.53,1.01]		0.88	[0.66,1.16]		0.88	[0.76,1.03]	
Schooling	1.16	[0.97,1.40]		1.07	[0.92,1.24]		1.03	[0.93,1.15]	
Live alone	1.73	[0.53,5.57]		1.43	[0.68,3.01]		2.38	[1.34,4.24]	∗∗
BMI categories									
Underweight	1.67	[0.47,5.89]		1.42	[0.72,2.82]		1.85	[1.09,3.14]	∗
Overweight	0.43	[0.12,1.54]		0.90	[0.36,2.27]		1.27	[0.63,2.53]	
Obese	0.92	[0.32,2.63]		1.14	[0.49,2.65]		1.24	[0.75,2.04]	
*N*	368			581			1,134		
BIC′	30.33			62.47			−42.91		

ADL: activities of daily living; IADL: instrumental activities of daily living; RRR: relative risk ratio; CI: confidence interval; BMI: body mass index.

^a^Remaining with disability is the reference category. Normal weight is the reference category for BMI, living accompanied is the baseline category for household arrangement, and never smoked is the reference category for smoking status.

***P < 0.001; **P < 0.05; *P < 0.10.

**Table 4:** Relative risk ratios of the impact of body mass index categories and body mass index changes on disability transitions, São Paulo, Brazil—2000–2006.

Variables	ADL		*P*	IADL		*P*	NAGI		*P*
	RRR^a^	95% CI		RRR	95% CI		RRR	95% CI	
*Incidence of disability (reference = remain free of disability) *									
Age	1.10	[1.07,1.13]	∗∗∗	1.11	[1.08,1.15]	∗∗∗	1.05	[1.01,1.09]	∗
Female	1.75	[1.04,2.94]	∗	1.53	[0.88,2.65]		2.44	[1.44,4.12]	∗∗
Smoking status									
Former smoker	0.85	[0.53,1.38]		1.14	[0.67,1.95]		1.47	[0.87,2.50]	
Current smoker	1.38	[0.71,2.70]		1.45	[0.67,3.16]		0.97	[0.56,1.69]	
Number of chronic conditions	1.56	[1.32,1.86]	∗∗∗	1.33	[1.12,1.59]	∗∗	1.25	[0.92,1.68]	
Schooling	0.97	[0.88,1.07]		0.92	[0.82,1.03]		0.90	[0.80,1.01]	
Live alone	0.85	[0.50,1.44]		0.60	[0.34,1.08]		1.01	[0.42,2.41]	
BMI categories									
Underweight	0.92	[0.48,1.77]		1.73	[1.05,2.84]	∗	0.93	[0.49,1.79]	
Overweight	0.72	[0.36,1.44]		1.39	[0.67,2.88]		1.87	[0.87,3.99]	
Obese	1.99	[1.10,3.57]	∗	2.38	[1.61,3.52]	∗∗∗	1.22	[0.66,2.27]	
BMI change									
Loss	1.23	[0.76,2.00]		0.99	[0.62,1.60]		0.85	[0.49,1.48]	
Gain	2.30	[1.03,5.12]	∗	1.97	[0.97,4.01]		2.15	[1.20,3.85]	∗
*N*	800			737			389		
BIC′	27.46			9.40			28.56		
*Recovery from disability (reference = remain with disability) *									
Age	0.92	[0.87,0.98]	∗	0.93	[0.88,0.99]	∗	0.95	[0.91,0.99]	∗
Female	0.43	[0.13,1.47]		0.41	[0.11,1.58]		0.53	[0.28,1.01]	
Smoking status									
Former smoker	0.34	[0.09,1.30]		1.40	[0.60,3.29]		1.46	[0.74,2.87]	
Current smoker	1.11	[0.29,4.17]		0.69	[0.10,4.70]		0.97	[0.38,2.44]	
Number of chronic conditions	0.64	[0.45,0.90]	∗	0.94	[0.68,1.29]		0.60	[0.46,0.80]	∗∗∗
Schooling	1.05	[0.87,1.28]		1.05	[0.82,1.34]		1.03	[0.87,1.22]	
Live alone	0.50	[0.12,2.02]		1.03	[0.22,4.85]		3.27	[1.63,6.57]	∗∗
BMI categories									
Underweight	0.49	[0.12,2.04]		0.16	[0.02,1.40]		0.71	[0.26,1.88]	
Overweight	0.89	[0.27,2.88]		0.76	[0.18,3.20]		0.86	[0.31,2.36]	
Obese	0.42	[0.17,1.05]		0.68	[0.22,2.07]		0.43	[0.21,0.86]	∗
BMI change									
Loss	0.52	[0.23,1.19]		0.53	[0.21,1.35]		1.07	[0.61,1.89]	
Gain	0.18	[0.05,0.68]	∗	0.64	[0.15,2.73]		0.53	[0.21,1.37]	
*N*	161			224			572		
BIC′	31.87			91.45			85.22		

ADL: activities of daily living; IADL: instrumental activities of daily living; RRR: relative risk ratio; CI: confidence interval; BMI: body mass index.

^a^Relative risk ratios were adjusted by smoking status. Remaining free of disability is the reference category for those who started without disability, and remaining with disability is the reference category for those who had disability in the baseline. Normal weight is the reference category for BMI. Stable weight is the baseline category for weight change. Results for lost in the followup were omitted (available upon request).

***P < 0.001; **P < 0.05; *P < 0.10.
